# Leadless vs. Transvenous Pacemakers in Patients with End-Stage Renal Disease: A Systematic Review and Meta-Analysis

**DOI:** 10.3390/biomedicines13081952

**Published:** 2025-08-09

**Authors:** Ștefan Bogdan, Mircea Ioan Alexandru Bistriceanu, Cosmin Gabriel Ursu, Andrei Constantin Anghel, Darie Ioan Andreescu, Alexandru Ababei, Silvia Deaconu, Alexandru Deaconu

**Affiliations:** 1 Faculty of Medicine, Carol Davila University of Medicine and Pharmacy, 050474 Bucharest, Romania; bogdan.stefan@umfcd.ro (Ș.B.); mircea-ioan-alexandru.bistriceanu0721@stud.umfcd.ro (M.I.A.B.); darie-ioan.andreescu0720@stud.umfcd.ro (D.I.A.); alexandru.ababei0125@rez.umfcd.ro (A.A.); si.deaconu@gmail.com (S.D.); alexandru.deaconu@umfcd.ro (A.D.); 2 Department of Cardio-Thoracic Pathology, Faculty of Medicine, Carol Davila University of Medicine and Pharmacy, 050474 Bucharest, Romania; 3 Elias Clinical Emergency Hospital, 011461 Bucharest, Romania; 4 Iliescu Emergency Institute for Cardiovascular Diseases, 022328 Bucharest, Romania; 5 Emergency Clinical Hospital, 014461 Bucharest, Romania; 6 Monza-Ares Hospital Bucharest, 021967 Bucharest, Romania

**Keywords:** leadless pacemaker, transvenous pacemaker, end-stage renal disease, dialysis, permanent pacemaker implantation

## Abstract

**Background**: Patients with end-stage renal disease (ESRD) are at elevated risk for device-related complications following pacemaker implantation. Leadless pacemakers (LPMs) offer theoretical advantages over transvenous pacemakers (TVPs), but their safety and efficacy in this high-risk population remain unclear. Our aim was to compare clinical outcomes and complication profiles between leadless and transvenous pacemakers in patients with ESRD. **Methods**: We conducted a systematic review and meta-analysis according to PRISMA guidelines, including three retrospective studies comparing LPMs and TVPs in ESRD patients. The primary endpoint was overall complications post-implantation. Secondary outcomes included early mortality (within 30 days), access site complications, device-related events, thrombotic events, and respiratory complications. A random-effects model was used to pool odds ratios (ORs) and 95% confidence intervals (CIs). **Results**: Three studies comprising 10.075 ESRD patients were included. No significant difference was found in overall complications (OR 1.35, 95% CI 0.78–2.33, *p* = 0.14) or early mortality (OR 1.01, 95% CI 0.42–2.43, *p* = 0.97) between LPM and TVP groups. However, LPMs were associated with increased access site complications (OR 2.51, 95% CI 1.06–5.90, *p* = 0.04), thrombotic events (OR 1.42, 95% CI 1.14–1.78, *p* = 0.03), and respiratory complications (OR 1.43, 95% CI 1.01–2.03, *p* = 0.05). Device-related complication rates were similar (OR, 1.09; 95% CI, 0.63–1.88; *p* = 0.30). Heterogeneity was low across most outcomes. **Conclusions**: Among patients with ESRD, leadless pacemakers did not reduce overall complications or short-term mortality compared to transvenous systems and were associated with increased risk of certain procedural complications. These findings could support a personalized approach to device selection in ESRD and highlight the need for further prospective studies to guide clinical decision-making in this population.

## 1. Introduction

Conventional transvenous pacemakers (TVPs) have long represented the standard of care for patients requiring permanent pacemaker implantation. However, their implantation involves endocardial leads and a subcutaneous pocket, both of which are associated with complications such as lead dislodgment, venous stenosis, tricuspid valve regurgitation, device infection, and long-term mechanical failure [1]. To mitigate these risks, leadless pacemakers (LPMs) were introduced as a fully intracardiac alternative, directly implanted in the right ventricle via a femoral venous approach. By eliminating leads and pockets, LPMs aim to reduce procedural complexity and hardware-related complications [2,3].

Recent comparative studies and real-world registries have supported the safety and efficacy of LPMs in the general population. Randomised and observational data indicate that LPMs are associated with reduced rates of pocket- or lead-related complications, including fewer infections, dislodgments, and mechanical lead failures [1,2,3,4,5,6,7,8,9,10]. LPMs have also shown favourable hemodynamic profiles, with lower incidence or progression of tricuspid regurgitation [1,11,12], reduced NT-proBNP levels [1], and stable pacing thresholds over time [4,5,6]. In a retrospective observational study from 2020, a lower incidence of pacing-induced cardiomyopathy (PICM) in leadless pacing systems compared to transvenous approaches was observed [12]. Nonetheless, concerns remain regarding procedural complications such as pericardial effusion, which may be more frequent in high-risk patients, especially in those with chronic kidney disease (CKD) or coagulopathy [7,8,9,13].

Despite these advantages, the clinical role of LPMs in patients with end-stage renal disease (ESRD) remains incompletely defined. This population presents a unique set of challenges, including higher risks of device infection, central venous stenosis, bleeding, and mechanical complications, as well as an overall poor prognosis and shorter life expectancy. Real-world studies have reported a higher use of LPMs in patients with ESRD compared to TVPs, driven by concerns about infection and the preservation of venous access [2,3,10,14,15,16]. This is reflected in current clinical guidelines; the 2021 European Society of Cardiology pacing and cardiac resynchronisation therapy guideline provides a Class IIb recommendation for the use of leadless pacemakers in patients at high risk of pocket infection or with limited venous access, including those on haemodialysis [17]. These patients still experience higher rates of in-hospital mortality, prolonged length of stay, and increased cost, likely due to their overall clinical fragility rather than the pacing modality itself [8,9,15]. Importantly, existing studies have not consistently provided ESRD-specific outcome data, and results across registries are heterogeneous. Therefore, a focused synthesis of available evidence comparing LPMs and TVPs in patients with ESRD is warranted to clarify their relative safety and performance in this high-risk group.

We performed a systematic review and meta-analysis to evaluate the potential benefits of leadless pacemakers compared to transvenous pacemakers on adverse complications after implantation in a fragile population with end-stage kidney disease.

## 2. Materials and Methods

**Figure 1:**
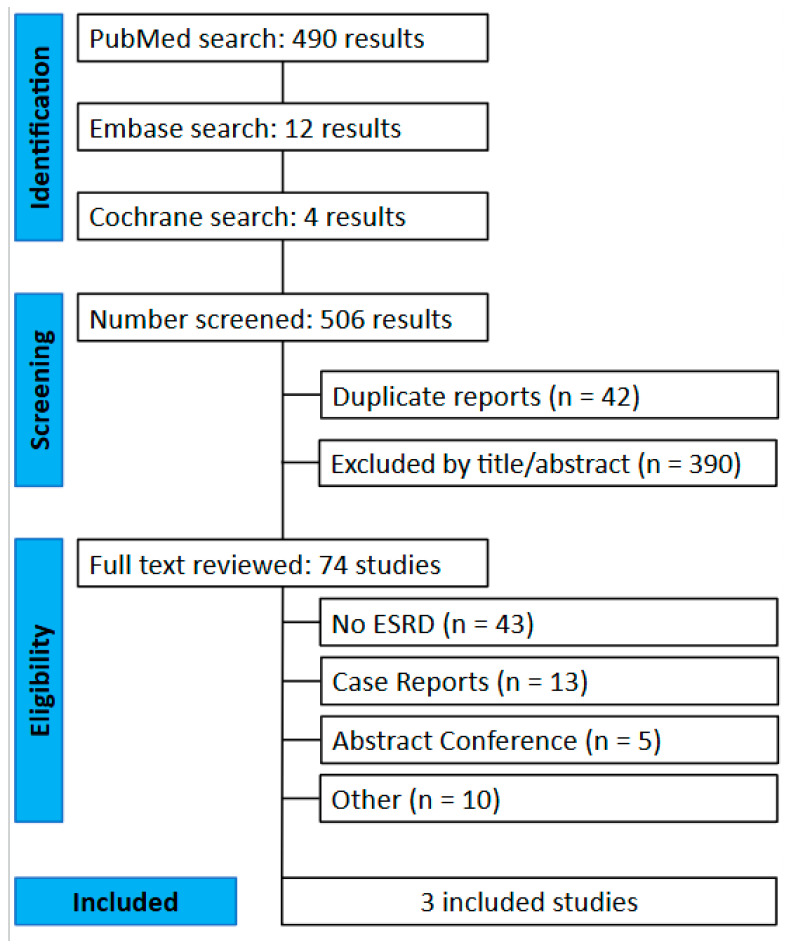
Flow diagram for material preparation and methodological framework of this study [18].

This systematic review and meta-analysis were conducted and reported per the Cochrane Collaboration Handbook for Systematic Review of Interventions and the Preferred Reporting Items for Systematic Reviews and Meta-Analysis (PRISMA) statement guidelines (Figure 1) [18].

### 2.1. Inclusion and Exclusion Criteria

Inclusion in this meta-analysis was restricted to studies that met all of the following eligibility criteria: (1) randomised clinical trials or observational studies, (2) evaluated the general complications and devices-related complications of leadless and transvenous pacemakers, (3) enrolled patients with end-stage kidney disease who required pacemaker implant, and (4) had a follow-up of at least 30 days. Additionally, studies were included only if they reported any of the clinical outcomes of interest. A minimum follow-up duration of 30 days was chosen based on a preliminary literature review, which revealed substantial heterogeneity in follow-up durations across different studies. We excluded studies with (1) interventions on animals, (2) no control group studies, (3) no reported outcomes of complications, (4) patients without stage 4 or 5 chronic kidney disease or requiring haemodialysis, and (5) no early mortality data reported.

### 2.2. Search Strategy

We systematically searched PubMed, Embase, and the Cochrane Central Register of Controlled Trials from inception to July 2025 using the following search strategy: (“ESRD” OR “Chronic kidney failure” OR “Chronic kidney failure” OR “CKF” OR “End-stage renal disease” OR “End-stage renal failure” OR “ESRF” OR “End-stage kidney failure” OR “Chronic kidney disease” OR “CKD” OR “Terminal kidney disease”) AND (“Artificial Pacemaker” OR “Cardiac Pacemaker” OR “Heart Pacemaker” OR “Transvenous Pacemaker” OR “Trans Venous Pacemaker” OR “Lead-based pacemaker” OR “Endocardial pacemaker” OR “Transvascular pacemaker” OR “Endovascular pacemaker” OR “Transvenous cardiac pacing system” OR “Lead-implanted pacemaker”) AND (“Leadless” OR “Wireless pacemaker” OR “Transcatheter pacemaker” OR “Self-contained pacemaker” OR “Miniaturized pacemaker” OR “Miniature intracardiac pacemaker” OR “Lead-free pacemaker”). The references of all included studies, previous systematic reviews, and meta-analyses were also manually searched for additional studies. The initial screening was independently performed by three authors (Ș.B., A.C.A., and M.I.A.B.), and full-text articles were retrieved when deemed potentially eligible by at least one reviewer. The same three reviewers independently assessed full texts for eligibility. Any disagreements were resolved by discussion, and if consensus could not be reached, a fourth reviewer (C.G.U.) acted as arbiter. No automation tools were used in the selection process. Data were extracted independently by two reviewers (A.C.A. and M.I.A.B.) using Microsoft Excel. If critical data were missing or unclear, the corresponding authors of the studies were contacted via email, with one follow-up reminder sent after two weeks. No automation tools were used in the data collection process.

The prospective meta-analysis protocol was registered on PROSPERO on 20 July 2025, under protocol #CRD420251109462.

Our objective was to analyse (1) the overall complications after pacemaker implant in ESRD patients as the primary endpoint for assessing the success of the intervention, alongside the following secondary endpoints: (2) the early mortality within 30 days, (3) the access site complications of PMI, (4) device-related complications, (5) cardiac thrombosis complications during hospitalisation, and (6) respiratory complications. Other variables that were collected were the study design, country, study duration, follow-up in days, age in years, patient sex distribution, number of patients with diabetes, number of patients with atrial fibrillation, number of patients with peripheral vascular disease, number of patients with chronic heart failure, number of patients with supraventricular arrhythmia, number of patients with ventricular arrhythmia, number of patients with tricuspid valve disease, and number of patients with pulmonary diseases.

### 2.3. Risk of Bias

We assessed the risk of bias using the Risk of Bias in Non-Randomised Studies of Interventions, version 2 (ROBINS-I V2) assessment tool. Two independent authors carried out the risk of bias assessment (A.C.A. and D.A.). Any disagreements were resolved through consensus after discussing the reasons for the discrepancy.

### 2.4. Statistical Analysis

The meta-analysis employed a therapeutic model. The endpoints were calculated as odds ratios and reported with 95% confidence intervals (CIs). To determine eligibility for each synthesis, we first tabulated the characteristics of each included study, including population, intervention, comparator, outcome, and time point. Only studies that provided sufficient data for the specific outcome of interest and aligned with the comparison groups were included in each meta-analysis.

A random-effects model was chosen for this analysis to account for both within-study sampling errors and between-study heterogeneity. This approach is recommended by Cochrane due to the prevalence of study design variability, making it nearly impossible to have methodologically identical studies. Between-study variance (τ^2^) was estimated using the restricted maximum-likelihood (REML) method, which remains a standard approach in random-effects meta-analyses due to its interpretability and robust performance across varying levels of heterogeneity. Heterogeneity was assessed using τ^2^, I^2^ (percentage of total variability attributable to heterogeneity), and H^2^ (ratio of total to within-study variability). Additionally, Cochran’s Q test was used to statistically evaluate the presence of heterogeneity. Because the Q test has limited power with smaller sample sizes, a significance level of 0.10 was used instead of the conventional 0.05, increasing sensitivity to detect true heterogeneity. A leave-one-out sensitivity analysis was performed when heterogeneity was considered at least moderate in order to assess the robustness of the results.

To assess the contribution of individual studies to the overall heterogeneity and their influence on the pooled effect size, a Baujat plot was generated using the meta package in R. The plot shows each study’s contribution to the total heterogeneity (*x*-axis) and its influence on the overall meta-analytic result (*y*-axis).

Publication bias was assessed using multiple complementary approaches. Funnel plots were generated to visually inspect asymmetry in the distribution of effect sizes against their standard errors. Afterwards, asymmetry tests (Egger’s test) were used to numerically evaluate publication bias.

Review Manager (RevMan, version 7.2.0 (Cochrane Centre, The Cochrane Collaboration, Copenhagen, Denmark) and R version 4.5.0 (R Foundation for Statistical Computing, Vienna, Austria)) were utilised for all statistical analyses.

## 3. Results

### 3.1. Study Selection and Baseline Characteristics

The initial search yielded 506 results. After removing duplicate records and ineligible studies, 74 remained and underwent full review based on the inclusion criteria. Of these, three studies were included, comprising 10.075 patients from three retrospective cohort studies (Figure 1). The LPMs patients had a mean age of 71.6 ± 11.2 years, of whom 40.1% were female, and an averaged median follow-up of 394 days. TVPs patients had a mean age of 72.6 ± 11 years, of whom 39.9% were female, and an averaged median follow-up of 90 days. Other baseline patient and study characteristics are detailed in Table 1.

### 3.2. Forest Plots

#### 3.2.1. Overall Complications After Pacemaker Implant

**Figure 2:**
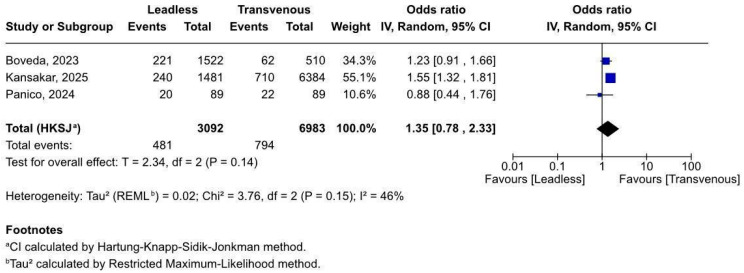
Forest plot of overall complications after pacemaker implantation in patients with end-stage renal disease [19,20,21].

Three studies (n = 10,075) reported on general complications after pacemaker implantation in ESKD patients. Meta-analysis (Figure 2) showed no significant difference between leadless and transvenous systems (OR 1.35, 95% CI 0.78–2.33, *p* = 0.14). Heterogeneity was moderate (I^2^ = 46%). A leave-one-out analysis was performed (Figure S1), showing Kansakar 2025 [20] as the sole source of heterogeneity; removing it reduced the I^2^ to 0%. Due to the limited number of retrospective studies and moderate variability, results should be interpreted with caution. Further randomised trials are needed to confirm these findings.

#### 3.2.2. Early Mortality Within 30 Days

**Figure 3:**
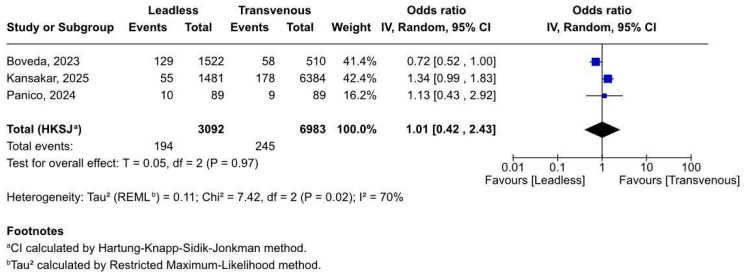
Forest plot of early all-cause mortality following pacemaker implantation [19,20,21].

Early all-cause mortality within 30 days post-procedure was reported in all three studies, encompassing 194 events in the leadless group and 245 in the transvenous group. Pooled analysis (Figure 3) showed no significant difference in short-term mortality between the two pacing modalities (OR 1.01, 95% CI 0.42–2.43), with a non-significant overall effect (*p* = 0.97).

However, the analysis was limited by high heterogeneity (I^2^ = 70%), reflecting inconsistencies across study populations and effect estimates. A leave-one-out analysis was performed (Figure S2), showing Kansakar 2025 [20] as the sole source of heterogeneity; removing it reduced the I^2^ to 0%. Notably, the wide confidence intervals preclude firm conclusions regarding relative safety in the early post-implant period. These findings highlight the need for more granular, event-level data and prospective trials specifically addressing early mortality in this high-risk population.

#### 3.2.3. Access Site Complications

**Figure 4:**
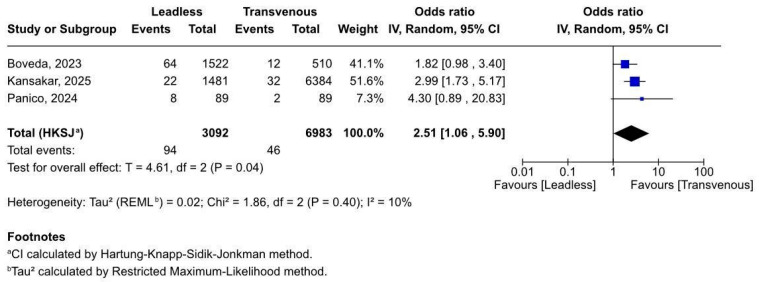
Forest plot of access site complications post-implantation [19,20,21].

Access site complications were significantly more frequent in patients receiving leadless pacemakers compared to those with transvenous systems (Figure 4). Across three studies (n = 10,075), the pooled odds ratio was 2.51 (95% CI 1.06–5.90), indicating more than double the risk in the leadless group (*p* = 0.04). Heterogeneity was low (I^2^ = 10%), supporting consistency across studies. Notably, all three studies showed a directionally consistent increase in events associated with the leadless approach, with two reporting statistically significant point estimates.

This finding highlights a potential procedural trade-off associated with femoral venous access in ESKD patients, who often present with challenging vascular anatomy and elevated bleeding risk. Procedural technique, operator experience, and device profile may all contribute and warrant further focused investigation. While statistically significant, this result should be interpreted as hypothesis generating, rather than a definitive answer. In order to accurately determine the true risk, targeted registries need to be made and published.

#### 3.2.4. Device-Related Complications

**Figure 5:**
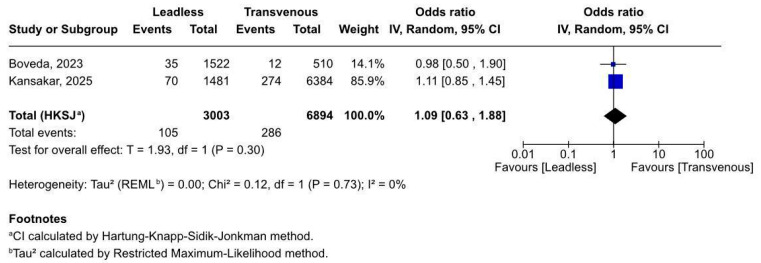
Forest plot of device-related complications after pacemaker implantation [19,20].

Two studies [19,20], totalling 9897 patients, evaluated device-related complications. No significant difference was observed (Figure 5) between leadless and transvenous pacemakers (OR 1.09, 95% CI 0.63–1.88, *p* = 0.30). The findings were statistically consistent (I^2^ = 0%, *p* = 0.73), with both studies reporting overlapping confidence intervals and neutral effect estimates.

Despite a numerically higher event rate in the transvenous group, the wide confidence intervals and lack of statistical significance indicate clinical equipoise between the two systems regarding device-related failures, malfunctions, or reinterventions.

#### 3.2.5. Cardiac Thrombosis Complications

**Figure 6:**
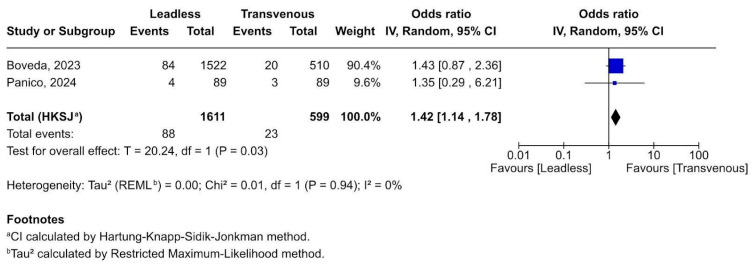
Forest plot of cardiac thrombotic events following pacemaker implantation [19,21].

Patients implanted with leadless pacemakers had 42% higher odds of developing thrombotic complications post-implant compared to those with transvenous pacemakers (Figure 6) This result is statistically significant (*p* = 0.03) and robust, with no evidence of heterogeneity (I^2^ = 0%). Both studies show point estimates > 1, favouring transvenous pacing in terms of thrombotic safety, although CIs overlap in individual studies.

#### 3.2.6. Respiratory Complications

**Figure 7:**
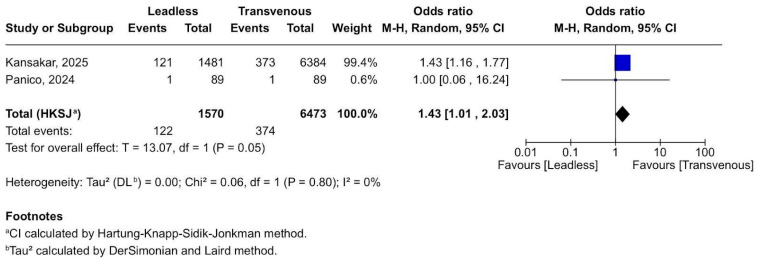
Forest plot of respiratory complications during hospitalisation after pacemaker implantation [20,21].

Leadless pacemakers were associated with 43% increased odds of in-hospital respiratory complications compared to transvenous pacemakers (Figure 7). This difference is statistically significant, although marginal (*p* = 0.05), and is supported by highly consistent data across studies (I^2^ = 0%). The majority of the effect comes from Kansakar et al. [20], given the weight of 99.4%, suggesting that this dataset is large and potentially more robust. While statistically significant, this result should be interpreted as hypothesis-generating, rather than a definitive answer. In order to accurately determine the true risk, targeted registries need to be made and published.

### 3.3. Quality Assessment

**Figure 8:**
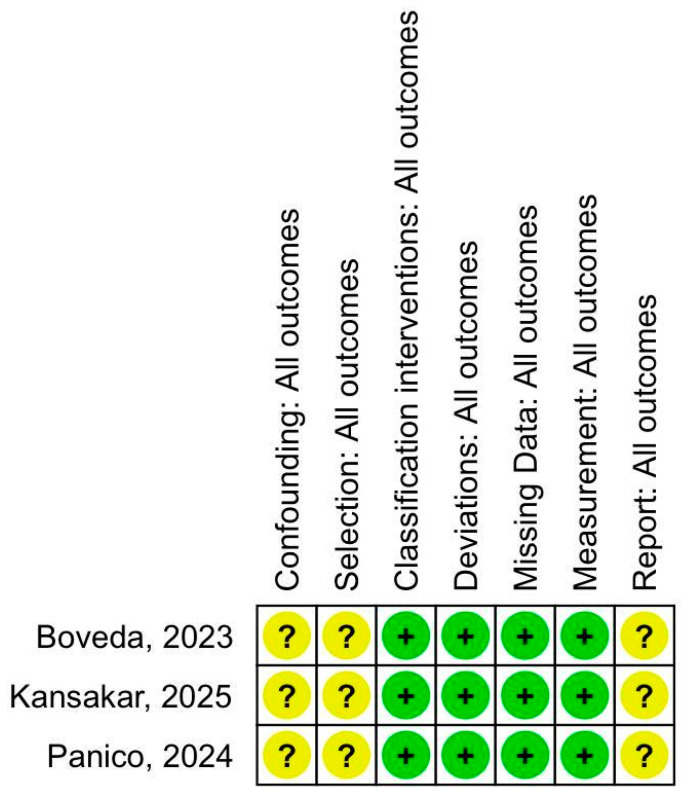
Quality assessment of the observational studies per Cochrane ROBINS-I [19,20,21].

The Cochrane Collaboration tool for assessing ROBINS was used for quality assessment. All retrospective studies were labelled as having a moderate risk of bias represented by the yellow question mark, while the low risk of bias domains were represented with a green plus sign, as described in Figure 8. The low number of studies within the primary outcome limited funnel plot analysis for publication bias. However, there was no significant asymmetry in the distribution of treatment effects with similar weights, which suggests no evidence of publication bias (Figure S3). Although the Egger test did not indicate a potential risk of bias (Table S1), it is not considered a reliable tool for meta-analyses with a relatively small number of studies. The Baujat plot did not reveal any studies with both high heterogeneity and high influence (Figure S4).

## 4. Discussion

In this systematic review and meta-analysis of over 10,000 patients with ESRD undergoing pacemaker implantation, we found no significant difference in overall complication rates or early all-cause mortality between leadless and transvenous pacemakers. However, leadless pacemakers were associated with significantly higher odds of access site complications, thrombotic events, and respiratory complications during hospitalisation. Device-related complication rates were statistically similar between the two groups. Notably, all analyses demonstrated low or no heterogeneity, except for early mortality, where between-study differences likely reflect variation in patient selection and clinical practice patterns.

Our findings suggest that while leadless pacemakers may offer theoretical advantages in ESRD patients, particularly by avoiding transvenous hardware and subcutaneous pockets, these benefits do not clearly translate into reduced overall complications or early mortality in this population. The increased rates of vascular and respiratory complications observed in the leadless group raise concerns about the procedural and post-procedural burden, especially in a patient population already prone to bleeding, thrombosis, and pulmonary vulnerability. In contrast, device-related failures and long-term hardware issues appear equivalent between the two systems, supporting clinical equipoise in this domain.

The observed excess in access-related and thrombotic complications in the leadless cohort may be attributed to several procedural factors, including the use of large-calibre femoral introducers (up to 27 Fr), prolonged sheath dwell time, and manipulation within the central venous and right heart circulation. Endothelial trauma and venous stasis may also contribute to increased thrombotic risk. Respiratory complications could reflect prolonged recumbency, sedation effects, or diaphragmatic irritation secondary to bleeding or effusion. It is also possible that LPM recipients in the included studies were clinically more fragile, with higher baseline comorbidity, which may confound these associations.

Our results align with multiple real-world registries and cohort studies suggesting that leadless pacemakers are frequently selected for ESRD patients due to perceived lower infection risk and ease of implantation [2,3,15,16]. However, consistent with the i-LEAPER registry and U.S. national analyses, our findings suggest that ESRD itself—not the pacing modality—is the dominant driver of poor outcomes [8,9,15]. Furthermore, the lack of mortality benefit contrasts with prior general-population studies such as Micra CED and AV CED, where LPMs demonstrated reduced device-related complications [3,7,13]. This discrepancy emphasises the importance of disease-specific evidence in high-risk populations.

A study by Mauriello et al. [22] presents a meta-summary of case reports describing the rare complication of implantable loop recorder (ILR) migration. Seven cases of ILR migration were identified, all occurring within 35 days post-implantation, suggesting early postprocedural vulnerability. Although migration mechanisms remain speculative, ranging from insertion angle and intercostal penetration to the effects of thoracic motion and negative pressure. The studies we analysed did not report this individual complication, rather it was included in the “device-related complications” section.

Several recent meta-analyses have consistently reported that leadless pacemakers are associated with favourable safety outcomes compared to transvenous systems in the general population. Gharehdaghi et al. [23], in an analysis of 21 observational studies and 24,597 patients, analysing complication incidence, infection rates, and all-cause mortality, found that leadless pacemakers were associated with significantly lower overall complication rates and fewer device-related infections, although all-cause mortality did not differ. Gangannapalle et al. [24], in a meta-analysis of 17 studies, comprising 36,168 patients, analysing total complications, all-cause mortality, and device-related complications, endocarditis, pneumothorax, required reintervention, and pericardial effusion confirmed these findings, highlighting reductions in endocarditis, pneumothorax, and reinterventions with leadless devices, albeit with a slightly increased incidence of pericardial effusion. Mhasseb et al. [25], in another large, pooled analysis comprised of 19 studies and 972,478 patients mostly from large observational studies, focusing on complication rate differences, similarly reported fewer mechanical and infectious complications with leadless systems but noted a significantly higher risk of cardiac tamponade. Habboush et al. [26] also demonstrated lower rates of infection and major complications with leadless pacemakers, while showing no difference in implantation success, pneumothorax, or vascular injury.

These conclusions were further strengthened by Ohn et al. [27], who conducted one of the largest meta-analyses to date, including 33,450 patients from 24 studies where the main endpoints were different types of complications, mortality and heart failure hospitalisation rates. They reported significantly lower rates of major complications, pulmonary adverse events, infections, and reinterventions in leadless pacemaker recipients, while also identifying a modest increase in vascular and cardiac injury complications. Mortality rates were again comparable between pacing modalities. Collectively, these data support the overall safety profile of leadless pacemakers in the general population.

It is important to note that none of these five meta-analyses included a focused analysis of patients with end-stage renal disease. In contrast, our meta-analysis specifically addresses this high-risk subgroup and finds a different pattern; while device-related complication rates were similar, leadless pacemakers were associated with increased access site, thrombotic, and respiratory complications, and offered no mortality advantage. These discrepancies likely reflect unique procedural vulnerabilities and baseline fragility in patients with end-stage renal disease, emphasising that general population trends cannot be directly extrapolated to specialised subgroups. Our findings highlight the potential of dedicated evidence when evaluating device safety and efficacy in complex populations.

Recent advances in leadless pacing aim to address the limitations of single-chamber VVIR systems. The AVEIR DR dual-chamber leadless pacemaker, studied in the AVEIR DR i2i trial, showed high implantation success and reliable atrioventricular synchrony across a broad patient population, representing a significant technological step forward [28]. These results are preliminary, and there is currently no specific data for patients with end-stage renal disease. Therefore, although dual-chamber leadless pacing is a promising development, its clinical significance for this vulnerable group remains uncertain.

Meanwhile, increasing evidence indicates that leadless pacemakers have a favourable safety profile regarding tricuspid valve function. A recent study by La Fazia et al. found that leadless devices were associated with a significantly lower incidence and progression of tricuspid regurgitation compared to transvenous systems [29]. This supports earlier research suggesting that avoiding a trans-tricuspid lead can reduce valve dysfunction, which is especially important for populations with a predisposition to right-sided heart disease.

These findings are clinically relevant because they challenge the previous assumption of superiority for leadless systems in ESRD. Future research should focus on patient-level predictors of benefit, procedural refinements (e.g., use of ultrasound guidance, smaller sheaths), and tailored anticoagulation strategies. Randomised trials and dedicated prospective registries in ESRD patients are needed to identify the ideal pacing modality for this population.

### 4.1. Limitations, Strengths, and Future Directions

#### 4.1.1. Limitations

This meta-analysis has several limitations. First, all included studies were observational and non-randomised, introducing potential bias from unmeasured confounding and selection effects. Although we employed a random-effects model and conducted heterogeneity assessments, unaccounted differences in patient profiles, procedural protocols, and operator expertise across centres may still influence the observed outcomes. Secondly, the number of studies available for each outcome was limited to only three, which restricted the power of our comparisons and limited the robustness of subgroup analyses. Notably, thrombotic and respiratory complications were reported in only two studies, requiring cautious interpretation despite statistical significance. Third, centre volume and institutional experience with leadless pacing may have varied widely across studies, particularly for early-generation devices, which could affect complication rates, such as perforation or vascular injury. Standardised procedural techniques and consistent operator training were not uniformly reported, which may impact generalizability. Fourth, outcome definitions and follow-up durations were inconsistent across studies. While all studies reported early complications and in-hospital outcomes, few provided standardised definitions for endpoints such as access site events or thrombotic complications, limiting comparability.

Frailty-related clinical indicators, including dialysis duration, serum albumin levels, and type of vascular access, were reported in only one of the included studies, preventing quantitative synthesis across the available evidence. The absence of these data in the majority of studies limits the ability to account for baseline differences in patient vulnerability, which may influence both treatment selection and outcomes. This raises the possibility of confounding by indication, whereby patients with greater frailty may have been preferentially selected for one device type over another. Such confounding could bias the observed associations and should be considered when interpreting the findings of the present meta-analysis.

An important limitation of first-generation leadless pacemakers is their restricted pacing mode. Most currently available systems operate in VVIR mode, which precludes atrioventricular synchrony and limits their use primarily to patients with permanent atrial fibrillation or paroxysmal atrioventricular block. In patients with sinus rhythm, right ventricular pacing without AV coordination may predispose to pacemaker syndrome or increase the risk of atrial fibrillation, as shown in previous trials comparing VVIR to DDDR pacing modes [30,31].

To address this, a dual-chamber leadless pacing system (AVEIR DR) was approved by the U.S. Food and Drug Administration (FDA) in 2023, offering the first wirelessly coordinated atrial and ventricular leadless pacing option [32]. While this represents a major technological advancement, cost remains a major barrier to widespread adoption. The estimated cost of the AVEIR DR system is approximately USD 24,000 per device, nearly double the cost of single-chamber leadless systems, which themselves are about four times more expensive than conventional transvenous dual-chamber pacemakers [33]. Economic considerations may influence clinical decision-making regarding leadless pacemaker implantation, especially with emerging devices. While systems such as Micra VR are associated with higher upfront cost, modelling studies suggest favourable cost-utility, with an estimated incremental cost of approximately USD 4277 and an incremental gain of 0.09 QALYs versus conventional transvenous systems—yielding an ICER of ~USD 47,379 per QALY, potentially acceptable in high-income settings [25]. Similarly, real-world data from a tertiary centre indicated that, despite higher procedural costs, leadless pacemakers reduced overall hospitalisation and complication-related expenditures when used after device extraction for infection [34]. These economic considerations raise questions about the cost-effectiveness of leadless pacing in routine clinical practice, particularly in healthcare systems with constrained resources. Overall, while leadless pacemakers may offer long-term economic advantages in select clinical settings, their adoption in general practice should carefully weigh both upfront device cost and potential savings from reduced complications, especially given the lack of cost-effectiveness data specifically in ESRD populations.

Although Micra CED is based on a registry linked to Medicare claims and NRD is an all-payer inpatient database, there may be overlap in the patient populations.

Finally, patient-level data were not available, which prevented the adjustment for relevant clinical variables, such as anticoagulation status, dialysis modality, frailty index, and vascular access history. This limitation is particularly relevant in the end-stage renal disease population, where the comorbidity burden is high and clinical trajectories are heterogeneous. Despite these limitations, the present meta-analysis provides a comprehensive synthesis to date, focusing exclusively on patients with end-stage renal disease, a population that has been historically underrepresented in device trials. All analyses demonstrated low heterogeneity, and findings remained consistent across sensitivity models, lending strength to the internal validity of the results.

#### 4.1.2. Strengths

This study provides the first focused meta-analysis evaluating leadless versus transvenous pacemakers specifically in patients with end-stage renal disease. By isolating this high-risk subgroup, we offer clinically relevant insights that complement and contrast with prior analyses performed in general populations. Another strength lies in the comprehensive and structured assessment of complications beyond device failure or infection, including thrombotic, respiratory, and vascular outcomes that are particularly pertinent in this population. The inclusion of contemporary real-world data sources increases the external validity of the results. Furthermore, heterogeneity across most endpoints was low or absent, suggesting consistency across datasets despite differences in geography, centre type, and patient selection. The study also adheres to PRISMA guidelines and employs rigorous risk-of-bias and statistical methodology.

#### 4.1.3. Future Directions

Future research should prioritise prospective, multicentre studies or dedicated registries capturing detailed procedural and post-procedural outcomes in patients with end-stage renal disease. These studies should stratify by dialysis status, vascular anatomy, anticoagulation regimens, and access site strategy to determine the optimal pacing modality in various clinical scenarios. Technological advancements such as dual-chamber leadless systems, improved delivery mechanisms, and integrated sensing algorithms may help mitigate procedural risks and expand the applicability of leadless pacing in fragile populations. However, these devices must be rigorously tested in populations with chronic kidney disease, including those on haemodialysis, before widespread adoption.

Finally, standardised reporting of complications, particularly thrombotic and vascular events, should be encouraged in future publications to enhance data comparability and allow more precise estimates of risk in vulnerable populations

## 5. Conclusions

In this systematic review and meta-analysis of patients with end-stage renal disease undergoing permanent pacemaker implantation, leadless systems were not associated with a reduction in overall complications or early mortality compared to transvenous pacemakers. While leadless devices showed no difference in device-related failure rates, they were associated with increased access site, thrombotic, and respiratory complications. These findings suggest that the choice of pacing modality in ESRD should be individualised, weighing potential procedural trade-offs against long-term hardware considerations. Further prospective, controlled studies are needed to clarify which subgroups may benefit most from a leadless approach and to optimise procedural techniques for this vulnerable population.

## Figures and Tables

**Table 1:** Summary of patient characteristics.

	Boveda, 2023 [19] (n = 2032)		Kansakar, 2025 [20] (n = 7865)		Panico, 2024 [21] (n = 178)	
Characteristics	Leadless (n = 1522)	Transvenous (n = 510)	Leadless (n = 1481)	Transvenous (n = 6384)	Leadless (n = 89)	Transvenous (n = 89)
Study design	Retrospective		Retrospective		Retrospective	
Country	United States		United States		France	
Study duration	2017–2019		2016–2021		2015–2021	
Follow-up (Days) *	728		30		728	
Age (Years) **	71.2 ± 11.2	75.5 ± 10.1	71.6 ± 11.1	72.3 ± 11.1	77.3 ± 9.8	77.9 ± 8.6
Female (n, %)	659 (43.3)	193 (37.8)	550 (37.1)	2566 (40.2)	31 (35.0)	27 (30.0)
Diabetes (n, %)	1213 (79.7)	389 (76.3)	1061 (71.6)	4579 (71.7)	68 (76.0)	66 (74.0)
Atrial fibrillation (n, %)	1018 (66.9)	419 (82.2)	420 (28.3)	1574 (24.7)	61 (69.0)	60 (67.0)
Peripheral vascular disease (n, %)	677 (44.5)	241 (47.3)	150 (10.1)	784 (12.3)	45 (51.0)	52 (58.0)
Chronic Heart Failure (n, %)	1182 (77.7)	415 (81.4)	867 (58.5)	3295 (51.6)	62 (70.0)	56 (63.0)
Supraventricular arrhythmia (n, %)	170 (11.2)	41 (8.0)	N/A	N/A	9 (10)	8 (9.0)
Ventricular arrhythmia (n, %)	302 (19.8)	91 (17.8)	N/A	N/A	3 (3.4)	4 (4.5)
Tricuspid Valve Disease (n, %)	508 (33.4)	204 (40.0)	N/A	N/A	13 (15)	11 (12.0)
Pulmonary Diseases (n, %) ***	607 (39.9)	205 (40.2)	369 (24.9)	1480 (23.2)	29 (33)	20 (22.0)

* The follow-up was reported as median. ** The age was reported as mean and standard deviation. *** The pulmonary diseases included were chronic obstructive pulmonary disease and pulmonary hypertension.

## Data Availability

This study is based on previously published data. No new data were generated or analysed.

## Supplementary Materials

The following supporting information can be downloaded at: https://www.mdpi.com/article/10.3390/biomedicines13081952/s1, Figure S1. Forest plot after leave-one-out of overall complications. Figure S2. Forest plot after leave-one-out analysis of early mortality within 30 days of pacemaker implant. Figure S3. Funnel plot for overall complications. Figure S4. Baujat plot for overall complications. Table S1. Egger’s regression test for overall complications.

## Abbreviations

The following abbreviations are used in this manuscript:

AV	Atrioventricular synchronous
CED	Coverage with evidence development
CI	Confidence interval
CKD	Chronic kidney disease
ESRD	End-stage renal disease
Fr	French (unit of catheter diameter)
LPM	Leadless pacemaker
NRD	National Readmissions Database
OR	Odds ratio
PICM	Pacing-induced cardiomyopathy
TVP	Transvenous pacemaker
