# Supplementary material for: Leadless vs. Transvenous Pacemakers in Patients with End-Stage Renal Disease: A Systematic Review and Meta-Analysis
Source: Biomedicines. 2025 Aug 9;13(8):1952. doi: 10.3390/biomedicines13081952 (PMC12383732; doi:10.3390/biomedicines13081952)
Supplement: Supplementary file 1 [file biomedicines-13-01952-s001.zip › biomedicines-3810761-supplementary.pdf]

Study or Subgroup	Leadless Events	Leadless Total	Transvenous Events	Transvenous Total	Weight	Odds ratio IV, Random, 95% CI
Boveda, 2023	221	1522	62	510	84.1%	1.23 [0.91 , 1.66]
Panico, 2024	20	89	22	89	15.9%	0.88 [0.44 , 1.76]
**Total (HKSJ[a])**		**1611**		**599**	**100.0%**	**1.16 [0.25 , 5.39]**
Total events:	241		84			

Test for overall effect: T = 1.26, df = 1 (P = 0.43)

Heterogeneity: Tau² (REML[b]) = 0.00; Chi² = 0.73, df = 1 (P = 0.39); I² = 0%

Odds ratio
IV, Random, 95% CI

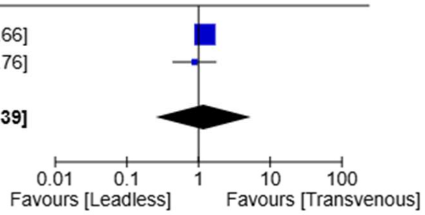

Footnotes

[a]CI calculated by Hartung-Knapp-Sidik-Jonkman method.

[b]Tau² calculated by Restricted Maximum-Likelihood method.

Figure S1. Forest plot after leave-one-out of overall complications

Study or Subgroup	Leadless Events	Leadless Total	Transvenous Events	Transvenous Total	Weight	Odds ratio IV, Random, 95% CI
Boveda, 2023	129	1522	58	510	89.4%	0.72 [0.52 , 1.00]
Panico, 2024	10	89	9	89	10.6%	1.13 [0.43 , 2.92]
**Total (HKSJ[a])**		**1611**		**599**	**100.0%**	**0.76 [0.13 , 4.29]**
Total events:	139		67			

Test for overall effect: T = 2.05, df = 1 (P = 0.29)

Heterogeneity: Tau² (REML[b]) = 0.00; Chi² = 0.75, df = 1 (P = 0.39); I² = 0%

Odds ratio
IV, Random, 95% CI

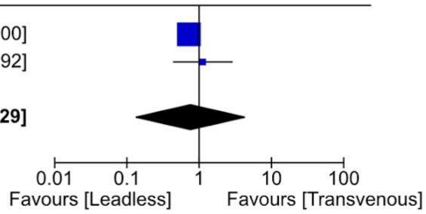

Footnotes

[a]CI calculated by Hartung-Knapp-Sidik-Jonkman method.

[b]Tau² calculated by Restricted Maximum-Likelihood method.

Figure S2. Forest plot after leave-one-out analysis of early mortality within 30 days of implant

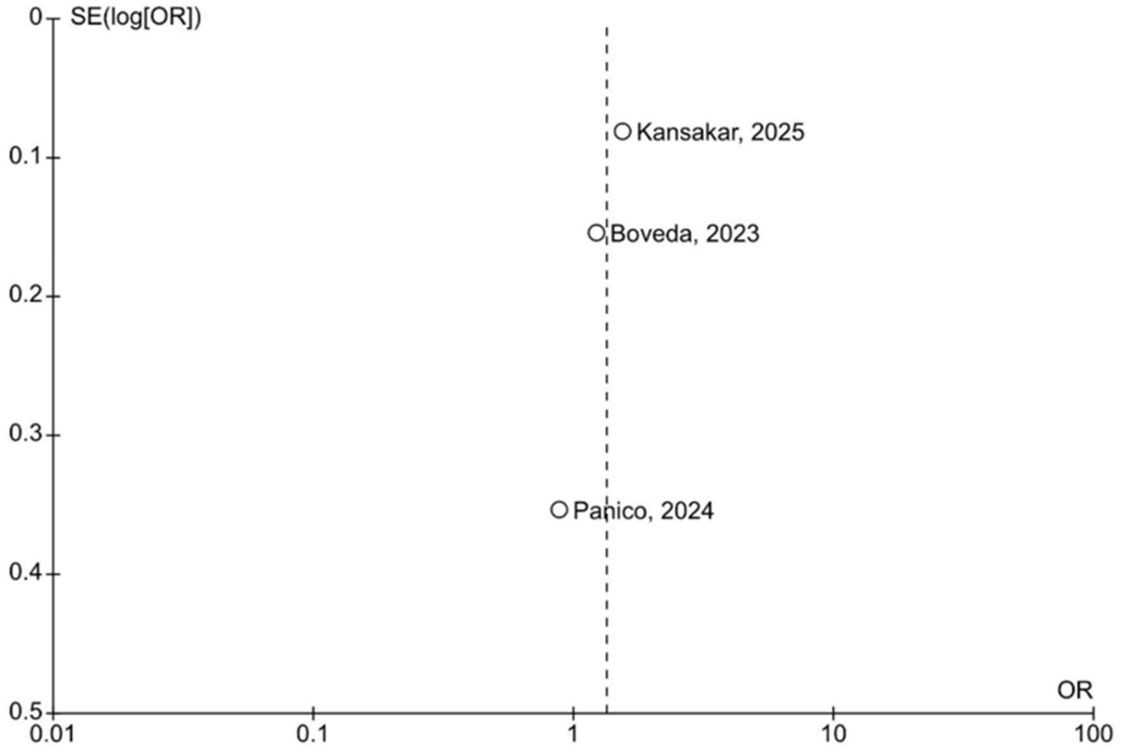

Figure S3. Funnel Plot for Overall Complications [18-20].

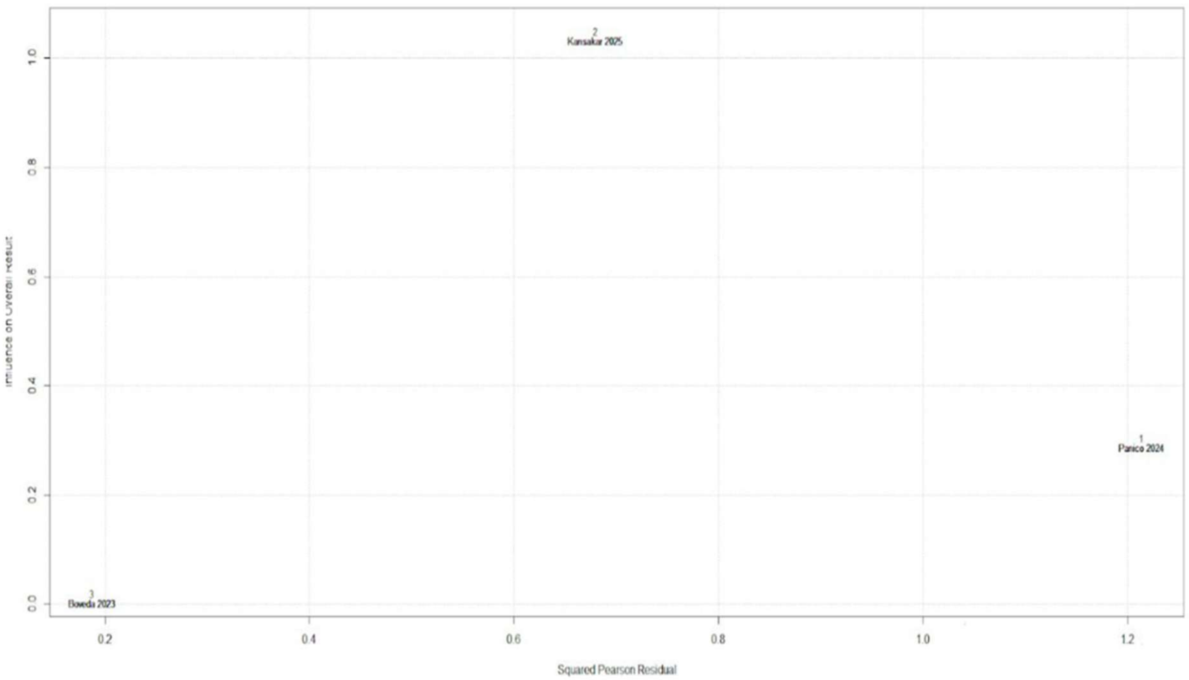

Figure S4. Baujat Plot for Overall Complications [18-20].

Parameter	Coef	SE	t	df	p	CI_lower	CI_Upper
Intercept	-3.87	0.869	-4.45	1	0.1406	-9.09	1.35
Limit estimate	0.61	NA	NA	NA	NA	-0.18	1.4

Table S1. Egger's Regression test for Overall Complications [18-20]
